# Micromechanical Properties of Nanostructured Clay-Oxide Multilayers Synthesized by Layer-by-Layer Self-Assembly

**DOI:** 10.3390/nano6110204

**Published:** 2016-11-08

**Authors:** Dongwei Hou, Guoping Zhang, Rohit Raj Pant, Zhongxin Wei, Shuilong Shen

**Affiliations:** 1 State Key Laboratory of Ocean Engineering and Collaborative Innovation Center for Advanced Ship and Deep-Sea Exploration (CISSE), Department of Civil Engineering, School of Naval Architecture, Ocean, and Civil Engineering, Shanghai Jiao Tong University, Shanghai 200240, China; houdw@sjtu.edu.cn; 2 Department of Civil & Environmental Engineering, University of Massachusetts Amherst, Amherst, MA 01003, USA; 3 Department of Civil and Environmental Engineering, Louisiana State University, Baton Rouge, LA 70803, USA; rpant1@alumni.lsu.edu (R.R.P.); weizhongxin@yahoo.com (Z.W.)

**Keywords:** layer-by-layer self-assembly (LbL), clay-based nanostructured multilayers, nanoindentation, atomic force microscopy, hardness, Young’s modulus

## Abstract

Clay-based nanostructured multilayers, such as clay-polymer multilayers and clay-oxide multilayers, have attracted growing attention owing to their remarkable mechanical properties and promising application in various fields. In this paper, synthesis of a new kind of nanostructured clay-oxide multilayers by layer-by-layer self-assembly was explored. Nano-mechanical characterization of 18 clay-based multilayer samples, prepared under as-deposited (i.e., air-dried) and annealing conditions at 400 °C/600 °C with different precursor cations and multilayer structure, were carried out using nanoindentation testing, atomic force microscopy (AFM), and X-ray diffraction (XRD). The influencing factors, including as-deposited and annealing conditions and clay concentrations on the mechanical properties were analyzed. Results show that all of the multilayers exhibit high bonding strength between interlayers. Higher modulus and hardness of clay-based multilayers were obtained with lower clay concentrations than that with higher clay concentrations. Different relationships between the modulus and hardness and the annealing temperature exist for a specific type of clay-oxide multilayer. This work offers the basic and essential knowledge on design of clay-based nanostructured multilayers by layer-by-layer self-assembly.

## 1. Introduction

Clay-based nanostructured multilayers (e.g., clay-polymer multilayers and clay-oxide multilayers [1,2,3,4]) have attracted growing attentions owing to their remarkable mechanical properties and promising application in various fields, such as protective coatings, micro/nanodevices, electronic memory structures and advanced optical and electro-optical devices, etc. [5,6,7]. The most advanced method for making clay-based multilayers is the layer-by-layer (LbL) self-assembly method [8,9,10,11,12,13,14], which is a bottom-up approach by sequential adsorption of nanometer-thick monolayers of oppositely-charged constituents (e.g., polyelectrolytes, charged nanoparticles, and biological macromolecules) to form a multilayered structure with nanometer-level control over the architecture [10,11]. The LbL self-assembly method, regarded as a surface precipitation process, is simple, versatile, low-cost, and low-temperature fabrication to produce nanostructured ultra-thin films with desirable properties.

Nanostructured clay-oxide multilayers prepared by the LbL self-assembly technique has been reported by Chen and Zhang et al. [15,16,17]. In these studies, the exfoliated clay platelet (montmorillonite, MMT) having a high aspect ratio with thickness in nanometer scale and bearing negative surface charges is used as an anionic component. The clay-zirconia multilayers were fabricated by sequentially dipping a Si/SiO_2_ substrate in the exfoliated nano-clay suspension and a zirconia cationic precursor solution followed by annealing at elevated temperatures. It was found that nanoscale LbL growth is achievable, and the annealed films remain uniform and crack-free. This indicates that a variety of nanostructured clay-oxide multilayers with graded functionalities can be conveniently manufactured via changing the composition or concentration of oxide cationic precursor solutions periodically or continuously.

The structural integrity and order of the nanostructured clay-oxide multilayers have been found to be of paramount importance for practical application. For example, a linearly increasing relationship generally exists between film thickness and number of adsorption cycles, which is reported by Chen and Zhang [15] and Fendler [18]. The degree of order in produced film varies widely, or even lack, any order at all in the self-assembled nanostructured materials [19]. Meanwhile, the thickness, morphology, and periodicity of the nanostructured film in different processing conditions has been characterized using a variety of techniques (both in situ and post-deposition), such as elipsometric measurements, light interference, adsorption isotherms, atomic force microscopy (AFM), scanning electron microscope (SEM), X-ray diffraction (XRD), X-ray reflectivity (XRR), transmission electron microscopy (TEM), and surface plasmon spectroscopic measurements, etc. [15,18,19,20,21,22,23].

To facilitate moving this promising research forward, the micromechanical properties, one of the most important aspects for a new material, of the clay-based nanostructured multilayers prepared under as-deposited (i.e., air-dried) and annealing conditions are investigated in this paper. Nanoindentation testing, assisted with atomic force microscopy (AFM) and X-ray diffraction (XRD), was employed to study the mechanical properties of these clay-oxide thin films. The influential factors, including the post-deposition condition and clay concentration, on the mechanical properties were analyzed, and issues with respect to design of desirable nanostructured clay-oxide multilayers are discussed. This work will offer the basic and essential knowledge on the design of clay-based nanostructured multilayers by LbL self-assembly.

## 2. Materials and Method

### 2.1. Sample Preparation

**Figure 1:**
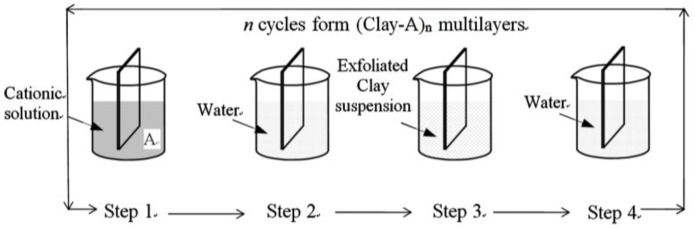
Schematic illustration of the clay-based nanostructured multilayer process (two-phase film). Step 1: adsorption of cations; step 2 and step 4: rinsing with water; step 3: adsorption of clay platelets.

All of the films in this paper, including (ZrO_2_)_30_, (ZrO_2_-MMT)_30_, (ZrO_2_-MMT)_60_, (SnO_2_-MMT)_30_, and (ZrO_2_-MMT-SnO_2_-MMT)_15_ multilayer films, were prepared via a layer-by-layer (LbL) deposition process as described in [15,17] (Figure 1). A NIMA^®^ automatic dip coater (Nima Technology Ltd., Coventry, UK) was used to deposit thin films. Epi-polished silicon wafers with thermally-oxidized surface layers (SiO_2_/Si) (Entegris Inc., San Diego, CA, USA), which is preferred for the wet chemical synthesis due to its relatively non-reactive SiO_2_ surface, were used as the substrate for all of the films.

Zirconia films were prepared by following a similar procedure described in [17]. The Si/SiO_2_ wafers were ultrasonically cleaned in 0.1 M NaOH solution for 20 min, immersed into piranha solution (3 vol. of 95–98 wt % H_2_SO_4_: 1 vol. of 30 wt % H_2_O_2_) for 20 min, rinsed with deionized water four times and dried in air. The substrates were alternately dipped into 0.06 M Zr^4+^ aqueous cationic precursor solution prepared from zirconium (IV) acetate hydroxide (Sigma-Aldrich, Saint Louis, MO, USA) and into 1.0 M aqueous ammonium hydroxide (NH_4_OH) anionic precursor solution at constant advance and withdraw dipping speeds of 20 mm per minute, with a holding time of 45 s in both anionic and cationic precursor solutions. Repeating the alternate deposition of 30 cycles gave an as-deposited (ZrO_2_)_30_ multilayered film, which was then isothermally annealed at 600 °C for 2 h in a box furnace, with ramped heating and cooling rates of 5 °C per minute.

A similar LbL deposition technique was employed to prepare clay-zirconia multilayered films [15,16]. Two kinds of sodium-rich montmorillonite (Na-MMT) clay minerals, Cloisite^®^ Na^+^, and SWy-2, obtained from Southern Clay Products Inc. (Gonzales, TX, USA) and the Clay Minerals Society Repository (Purdue University, West Lafayette, IN, USA), respectively, were used to prepare 0.03 wt % and/or 0.4 wt % clay suspension. The clay samples were first stirred with magnet at 1000 rpm speed in deionized water for 20 min and then ultrasonicated for 20 min to achieve complete dispersion and exfoliation. The pre-cleaned substrates were dipped into a 0.1 M zirconium cationic precursor solution (pH = 4.14) prepared from zirconium (IV) acetate hydroxide and into the exfoliated clay suspension alternately without inter-dipping rinsing. Both the dipping and withdrawing speeds were controlled at 20 mm per minute. The holding time was kept to 45 s in air, Zr precursor solution, and clay suspension. The deposition cycle was repeated 30/60 times to prepare a (clay-ZrO_2_)_30/60_ multilayered film. The substrate was then air-dried at room temperature to form the as-deposited (clay-ZrO_2_)_30/60_ films, and further annealed isothermally to 400/600 °C for 2 h at a constant heating and cooling rate of 5 °C per minute to form the annealed (clay-ZrO_2_)_30/60_ films. Clay-tin oxide films (SnO_2_)_30_ and (ZrO_2_-Clay-SnO_2_-Clay)_15_ multilayer films were prepared with the same method as clay-zirconia films, with a clay solution of 0.4 wt % and cationic precursor solution of 0.05 M SnCl_2_. Summary of the clay-oxide films studied in this paper is presented in Table 1.

### 2.2. Nanoindentation Testing

Nanoindentation experiments were performed using an MTS Nano XP indenter^®^ (MTS Nano Instruments Inc., Oak Ridge, TN, USA) at room temperature (~24 °C). A regular XP head and a dynamic contact module (DCM) head equipped with a diamond Berkovich indenter tip with a radius of <20 nm were used to indent the samples under a continuous stiffness measurement (CSM) mode, with load and displacement resolution of 50 nN and <0.01 nm for XP mode and 1 nN and <0.00002 nm for DCM mode. Combining a high-resolution DCM mode and a regular XP mode for shallow and deep indentations, respectively, improves the reliability of the test results.

Before indentation testing, each film, together with its substrate, was glued onto a cylindrical aluminum puck using melted Crystalbond 509 amber resin (Aremco Products Inc., Valley Cottage, NY, USA) heated to 130 °C. For each sample, a rigorous five-step testing scheme was employed to ensure high reliability and accuracy of the results: (1) tip cleaning by indentations (nine indents) on a piece of Scotch double-sided sticky tape; (2) pre-testing of the tip by indenting on standard fused silica to calibrate the tip and check the instrument working status; (3) film indentation testing on the film sample under XP and/or DCM mode at locations (15 indenting locations for each mode with a 3 × 5 array) spaced at 150 μm apart for XP mode, and in the same pattern but with 0.5 spacing shift for DCM mode; (4) post-testing tip checking by indenting on the same standard fused silica again. If the measured modulus and hardness value deviated from the standard value significantly, the data obtained in Step 3 were discarded and a new measurement was made starting with Step 1; and (5) data screening by checking the agreement of the 15 load-displacement curves plotted together. If a large discrepancy existed, all the data were discarded and indentations were repeated by selecting a new area of the film. If one or two curves shifted away from the majority curves, these curves were discarded and the rest of the data were regarded as acceptable.

**Figure 2:**
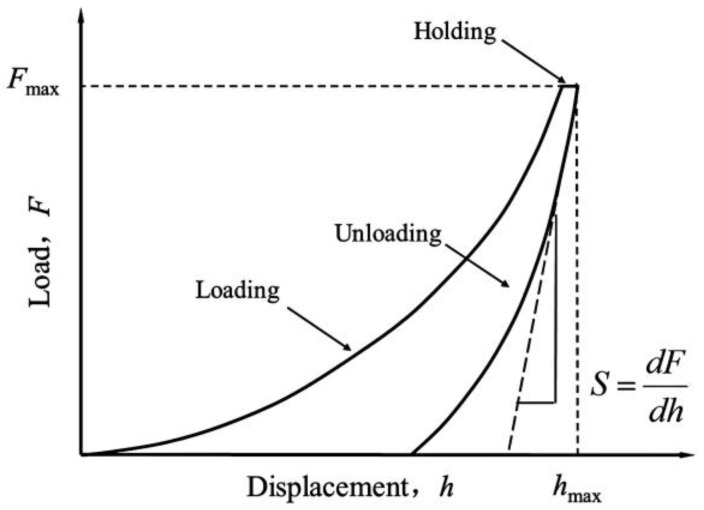
Schematic illustration of nanoindentation loading and unloading processes.

The XP and DCM indentations followed the ISO 14577 method [24] under the load control mode, since no significant creep occurred within the peak load holding period during the preliminary indentation tests. For the 15 indentations under XP mode, the maximum load *F*_max_ ranged from 500 mN to 0.39 mN, with each subsequent *F*_max_ being 60% of the previous value. The same loading scheme was also used for the 15 DCM indentations, except that *F*_max_ ranged from 10 mN to 0.008 mN. For both modes, a trapezoidal loading profile was used for all indentations, consisting of five steps (Figure 2): (1) increase the load linearly to a specified maximum value (*F*_max_) at a constant loading rate with a loading time of 30 s; (2) hold *F*_max_ constant for 10 s; (3) decrease the load linearly to 10% of *F*_max_ at the same rate as loading; (4) hold the load constant for 60 s to record the thermal drift of the instrument; and (5) decrease the load to zero linearly at the same loading rate in Steps (1) and (3).

### 2.3. Data Analysis Method

The Young’s modulus was derived from the load *F*, indentation depth *h*, and harmonic contact stiffness *S* recorded during the loading section of the test by using the Oliver and Pharr method [22,23,25,26]. An elastic parameter, reduced modulus (*E_r_*), is obtained by:

(1) $$E_{r}=\frac{\sqrt{\pi }}{2\beta \sqrt{A_{c}}}S$$

where β is a dimensionless correction factor for the indenter tip shape and β = 1.05 is commonly recommended for a Berkovich indenter [26]; *S* is the contact stiffness defined as the slope of the initial unloading curve at the maximum indentation depth (*h*_max_); *A_c_* is the projected contact area between the indenter and sample, which is obtained through the contact depth *h_c_* [22,23]:

(2) $$A_{c}=C_{0}h_{c}^{2}+\sum_{j=1}^{8}C_{j}h_{c}^{{\left(\frac{1}{2}\right)}^{j-1}}$$

where *h_c_* can be determined by:

(3) $$h_{c}=h_{\max}-\varepsilon \frac{F_{\max}}{S}$$

where ε is a constant that depends on the indenter tip geometry (for a Berkovich tip, ε = 0.75). For a perfect Berkovich indenter, the leading term *C*_0_ is 24.5; *C_j_* (*j* = 1, 2, …, 8) are needed to describe the deviations of the tip shape from the perfect Berkovich geometry owing to tip blunting. These constants were determined through tip calibration indentation tests on standard fused silica with a Young’s modulus *E* of 72 GPa.

The elastic modulus of the sample is derived by [27,28]:

(4) $$\frac{1}{E_{r}}=\frac{1-\nu_{c}^{2}}{E_{c}}+\frac{1-\nu_{i}^{2}}{E_{i}}$$

where ν_c_ and ν_i_ are the Poisson’s ratio of the sample and indenter; *E_i_* and *E_c_* are the elastic modulus of the indenter and sample, respectively. For a diamond indenter, *E_i_* = 1141 GPa, ν_i_ = 0.07. For the case of thin films, in which the indented zone is affected by the underlying substrate, *E_c_* represents the “mean” response of the film-substrate composite.

Indentation hardness, also regarded as the composite hardness for substrate-affected films, is determined by:

(5) $$H_{c}=\frac{F_{\max}}{A_{c}}$$

The Oliver and Pharr method has been widely used to determine the hardness and elastic modulus of small-scale bulk materials. It can also be used for thin films on substrate, provided that the maximum indentation depth is less than 10% of the film thickness to avoid the stiffness contribution of the substrate to the indenter-sample contact [15]. Much effort has been devoted to finding methods of deriving the film modulus and hardness from the measured composite response of tip-film-substrate system, e.g., introduction of the exponential weighting factors to divide contributions of the film and substrate to the composite modulus by Doerner et al., King et al. and Saha et al. [28,29,30]; a closed-form solution based on moduli-perturbation method by Gao [31]; an extrapolating method of the best-fit curve of experimental data by Mencik et al. [32] and Fisher-Cripps [33,34]; the deconvolution of film properties method modified from Hu’s solution [35] by Jung et al. [36]. In this paper, the method proposed by Korsunsky et al. [37] and Tuck et al. [38] based on an energy-based analysis of indentation testing is used to extract the film hardness *H_f_* by:

(6) $$H_{c}=H_{s}+\frac{H_{f}-H_{s}}{1+{\left[\left(h/t\right)/\beta_{0}\right]}^{X}}$$

Similarly, the film modulus *E_f_* can be obtained by the following equation proposed by Wei and Zhang [39,40]:

(7) $$E_{c}=E_{s}+\frac{E_{f}-E_{s}}{1+{\left[h/\left(t\beta_{1}\right)\right]}^{Y}}$$

where β_0_, β_1_, *X* and *Y* are constants obtained through curve-fitting of the experimentally-determined *H_c_* and *E_c_*; *H_s_* and *E_s_* are the substrate hardness and modulus, respectively, with *E_s_* = 172.4 GPa and *H_s_* = 12.28 GP for the Si/SiO_2_ wafer substrate used herein; *h*/*t* is the normalized indentation depth, a non-dimensional parameter. In the present study, the film-substrate composite elastic modulus *E_c_* and composite indentation hardness *H_c_* were obtained using the nanoindenter control program TestWorks Explorer^®^ based on the Oliver and Pharr method [22,23] with an assumed Poisson’s ratio of 0.22 for all of the films, where the value of the Possion’s ratio has little influence on the indentation results [30,32]. Deconvolution of film properties from composite hardness and modulus using Equations (6) and (7) was achieved by the statistical analysis program SAS^®^ version 9.1, where nonlinear procedures and Newton iterative methods were adopted. It is noteworthy that the inaccuracy of film thickness *t* used in normalizing indentation depth (*h*/*t*) only causes the shift of the fitted curve along the *h*/*t* axis, but does not induce errors to the fitted film hardness *H_f_* and modulus *E_f_*. This advantage avoids the time-consuming work on the determination of film thickness. Another advantage is that the fitted substrate *E_s_* and *H_s_* can be used to check the reliability and accuracy of the experiment data, since they can be easily obtained from literature or can be accurately determined by nanoindentation.

### 2.4. Atomic Force Microscopy (AFM) Images

The surface morphology of the films was characterized using a 5500 Scanning Probe Microscope (Agilent Technologies, Inc., Chandler, AZ, USA) with scan resolution of 512 pixels × 512 pixels, set point 1.000 V, and scan rate 1.0 Hz. The measurement was conducted in ambient temperature, in a contact mode with the PointProbe^®^Plus silicon tip (Nanosensors, Neuchatel, Switzerland) of 10–15 µm in height. The most commonly used surface roughness parameter *R_a_* is used to characterize the surface topography:

(8) $$R_{a}=\frac{1}{n}\sum_{i=1}^{n}\left|y_{i}\right|$$

where *y_i_* is the vertical distance from the mean line to the *i*th data point; *n* is the total tracing number within a specific area.

The residual indent image scanned by AFM is used to characterize the indent behavior (e.g., pile-up/sink-in, delamination between layers and between film and substrate) in parallel with the corresponding load-displacement curve.

### 2.5. X-ray Diffraction (XRD)

Expandability of the clay-based multilayers formed under as-deposited and annealed conditions was investigated by X-ray diffraction (XRD). XRD patterns were collected on a Siemens D-5000 diffractometer using Cu-Kα radiation generated at 40 kV and 30 mA and a scan range of 2°–34° (2θ). All scans employed sample spinning and a 0.996° divergence slit, 0.501° scatter slit, 0.1 mm receiving slit, a speed of 1° (2θ)/min, and a step size of 0.02° (2θ).

## 3. Results and Analysis

### 3.1. Typical F–h Curves and Residual Images

**Figure 3:**
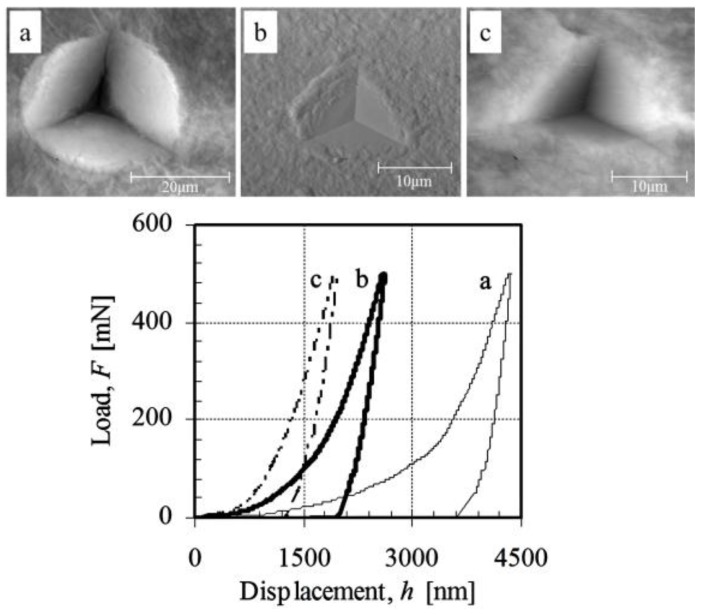
AFM images of typical indent impressions of (**a**) as-deposited; (**b**) annealed at 400 °C; and (**c**) annealed at 600 °C for 2 h (SnO_2_-MMT)_30_ multilayers with MMT 0.4 wt % and corresponding load-displacement curves. (AFM: atomic force microscopy; MMT: montmorillonite)

**Figure 4:**
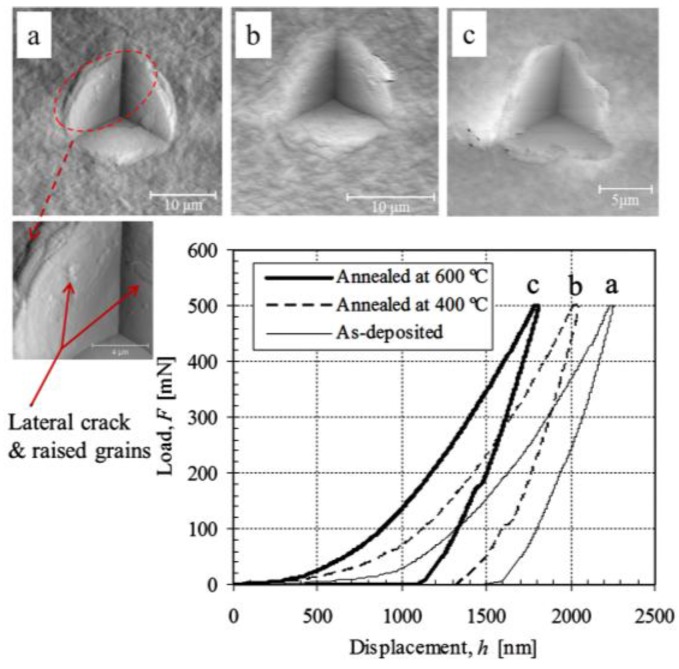
AFM images of typical indent impressions of (**a**) as-deposited; (**b**) annealed at 400 °C; and (**c**) annealed at 600 °C for 2 h (ZrO_2_-MMT-SnO_2_-MMT)_15_ multilayers with MMT 0.4 wt % and corresponding load-displacement curves.

The typical indentation load-displacement curves and indent impressions of (SnO_2_-MMT)_30_ and (ZrO_2_-MMT-SnO_2_-MMT)_15_ multilayers are shown in Figure 3 and Figure 4, respectively. The total indentation depth and residual depth under the same maximum load are greatly reduced as the annealing temperature increases for both multilayers. The indent images show that an apparent pile-up occurred around the contact impressions for the as-deposited films. A tendency of sink-in impressions increases with the increment of the annealing temperature, which makes the film become stiffer. No visible radial cracks developed for all the samples as that happened in muscovite, a naturally-formed nanostructured multilayer [40,41]. A partial lateral crack and a few raised grains on the surface of impression are observed in the AFM image shown in insert (a) of Figure 4. These grains with dimension of 300–600 nm are possibly the non-exfoliated clay particles.

Smooth loading curves and non-visible cracks in Figure 3 and Figure 4 demonstrate that the bonding strength either between clay platelets and cation layers or at the interface between films and substrate is strong enough to resist the complicated stress and strain fields generated by the sharp indenter. The film thickness of annealed films at 600 °C considerably shrinks by ~50% compared with that of the as-deposited condition [15]. Such structural integrity may be attributed to the high lateral bond strength of clay platelets, as well as high bonding strength. It is evident that clay-oxide multilayers prepared by the above method have a great advantage of superior tolerance to mechanical strain.

**Figure 5:**
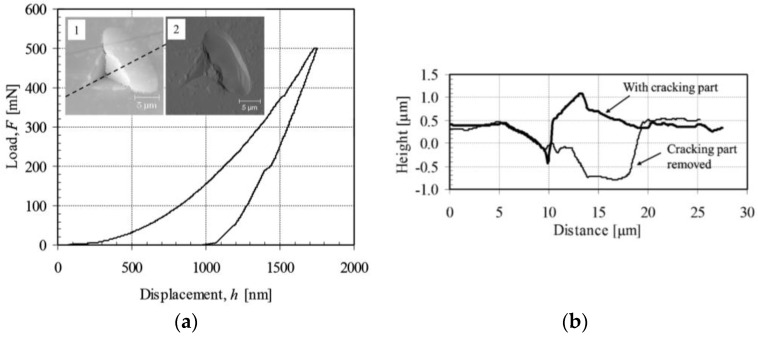
AFM images of typical indent impression of as-deposited (ZrO_2_-MMT)_30_ multilayers with MMT 0.03 wt % (**a**) and corresponding cross-section profiles (**b**).

An AFM image of one indent impression of as-deposited (ZrO_2_-MMT)_30_ multilayers with MMT 0.03 wt % was taken after 4 months of indentation testing and is shown in insert (1) of Figure 5a. The corresponding *F*–*h* curve indicates that small cracks occurred during both loading and unloading processes. However the jumped displacement under relatively constant load shown by the *F*–*h* curve is far smaller than that measured by the AFM images shown in the cross-section profile. This is mainly due to the continuous recovery of the strain within the indentation-affected volume during the long unloading time, which makes the cracked part rise upwards. Further investigation of the indent impression is conducted by carefully removing the cracked part and taking an AFM image, which is shown in insert (2) of Figure 5a. The cross-section profile along the dashed line in insert (1) of Figure 5a is shown in Figure 5b, indicating that the deepest cracking surface occurred far below the residual apex level. This reveals that the crack has passed the interface between the film and substrate and propagated into the substrate. Smoothness of the exposed crack surface further manifests that no delamination appears either between the multilayers or at the interface between the multilayers and the substrate.

### 3.2. Effect of As-Deposited and Annealing Procedure

**Figure 6:**
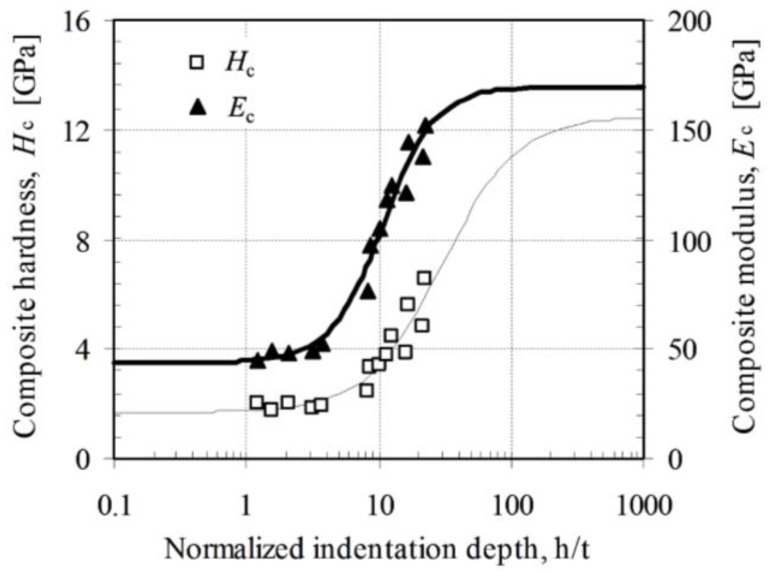
Example of film modulus and hardness fitting on experimental data of (ZrO_2_-MMT)_30_ annealed at 600 °C with clay montmorillonite 0.4 wt %. Fitted *E_f_* = 43.75 ± 4.86 GPa, *E_s_* = 169.60 ± 7.83 GPa, *H_f_* = 1.68 ± 0.36 GPa, and *H_s_* = 12.50 ± 0.56 GPa.

**Figure 7:**
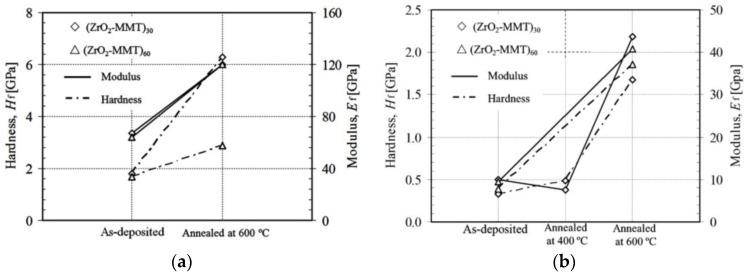
Hardness and modulus of zirconia-clay multilayers under as-deposited and annealed at 400 °C/600 °C conditions with clay montmorillonite 0.03 wt % (**a**) and 0.4 wt % (**b**).

Figure 6 shows an example of experimental data fitting result using statistical analysis program SAS^®^ version 9.1.3 on the zirconia-MMT multilayers. A summary of fitted film hardness and moduli of all of the testing samples is presented in Table 1, among which the typical results of the as-deposited and annealed conditions (at 600 °C) (ZrO_2_-MMT)_30_ and (ZrO_2_-MMT)_60_ multilayers with 0.03 wt % and 0.4 wt % of clay suspension are shown in Figure 7. It is evident that the annealing process considerably enhances the film hardness and modulus. For the zirconia-MMT multilayers with 0.03 wt % of clay suspension (Figure 7a), *H_f_* increases from 1.82 GPa to 6.29 GPa for (ZrO_2_-MMT)_30_, and from 1.67 GPa to 2.89 GPa for (ZrO_2_-MMT)_60_. Meanwhile, significant increment of moduli *E_f_* of the annealed multilayers (from 67.06 GPa to 119.9 GPa for (ZrO_2_-MMT)_30_, and from 63.78 GPa to 120.1 GPa for (ZrO_2_-MMT)_60_) were observed compared with the as-deposited ones. For the zirconia-MMT multilayers with 0.4 wt % of clay suspension (Figure 7b), *H_f_* increases from 0.33 GPa to 1.68 GPa for (ZrO_2_-MMT)_30_, from 0.39 GPa to 1.86 GPa for (ZrO_2_-MMT)_60_, and *E_f_* increases from 9.94 GPa to 43.75 GPa for (ZrO_2_-MMT)_30_, from 9.36 GPa to 40.69 GPa for (ZrO_2_-MMT)_60_. One reason for such enhancement of hardness and moduli is that the annealing process strengthens the bonding interaction between clay layers and Zr^4+^ cationic layers, where excess water is expelled out by physical and chemical reactions. Consequently, the interlayer spacing of clay layers occupied by Zr^4+^ cationic layers is reduced, resulting in a much thinner thickness of annealed multilayers than the as-deposited one. This is manifested by the average film growth rates of as-deposited and annealed multilayers of ~6.8 nm and 3.2 nm, respectively [15].

### 3.3. Effect of Annealing Temperature

**Figure 8:**
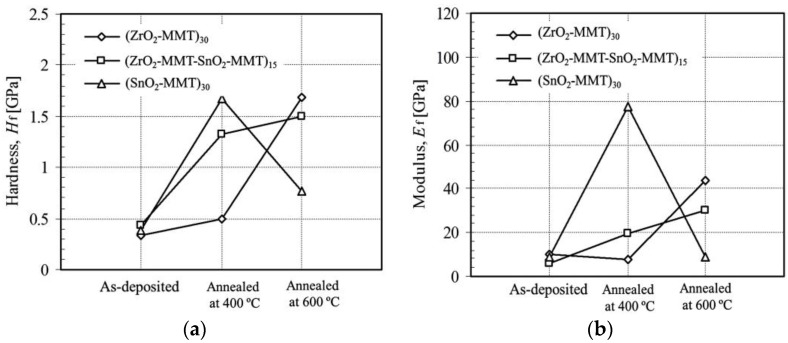
Hardness (**a**) and modulus (**b**) of zirconia-clay multilayers under as-deposited and annealed at 400/600 °C conditions with clay montmorillonite 0.4 wt %.

Annealing process was applied at 400 °C and 600 °C on the three different multilayers (ZrO_2_-MMT)_30_, (ZrO_2_-MMT-SnO_2_-MMT)_15_, and (SnO_2_-MMT)_30_. Figure 8 shows the fitted hardness (Figure 8a) and modulus (Figure 8b) of the films formed under as-deposited, annealed at 400 °C, and annealed at 600 °C conditions. In the as-deposited deposition condition, all three multilayers have almost the same hardness (0.33 GPa, 0.43 GPa and 0.43 GPa) and slightly different moduli (9.94 GPa, 6.2 GPa and 8.8 GPa). However, significant difference of hardness and moduli was observed as annealing temperature varies. (ZrO_2_-MMT)_30_ film exhibits the highest hardness and modulus at an annealing temperature of 600 °C among these three formed conditions, whereas (SnO_2_-MMT)_30_ film reaches its highest hardness and modulus at a temperature of 400 °C, then drops down sharply at 600 °C, much lower than those of (ZrO_2_-MMT)_30_ multilayers. The hardness and modulus of (ZrO_2_-MMT-SnO_2_-MMT)_15_ reasonably resides between those of (ZrO_2_-MMT)_30_ and (SnO_2_-MMT)_30_. This implies that there exists a relationship between an annealing temperature and a corresponding hardness and modulus for a specific clay-oxide multilayer.

### 3.4. Effect of Clay Concentration

**Figure 9:**
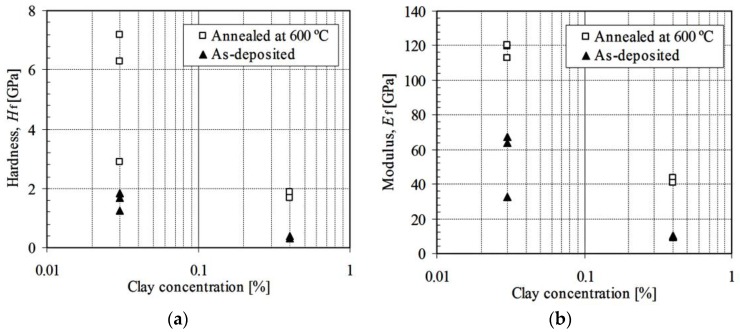
Effect of clay suspension concentration on hardness (**a**) and modulus (**b**) of zirconia-clay multilayers under as-deposited and annealed at 600 °C conditions.

Figure 9 shows the hardness (Figure 9a) and modulus (Figure 9b) of clay-zirconia multilayers (30 and 60 deposition cycles) formed by the LbL procedure with two different clay suspension concentrations of 0.03 wt % and 0.4 wt %. The films with lower clay concentration exhibit much higher hardness and moduli than those with higher clay concentration for both as-deposited and annealed (at 600 °C) multilayers. This indicates that high clay concentration does not facilitate the increment of hardness and modulus of multilayers. One reason is that the higher clay concentration generates a higher film growth rate of 37 nm/cycle [15], which is much higher than 5.7 nm/cycle corresponding to a clay concentration of 0.03 wt %, subsequently creating higher porosity within the multilayers. In addition, each thicker individual clay layer constructs a strong thermal barrier which inhibits the effectiveness of the annealing process across the whole film thickness.

### 3.5. Expandability vs. Forming Conditions

**Figure 10:**
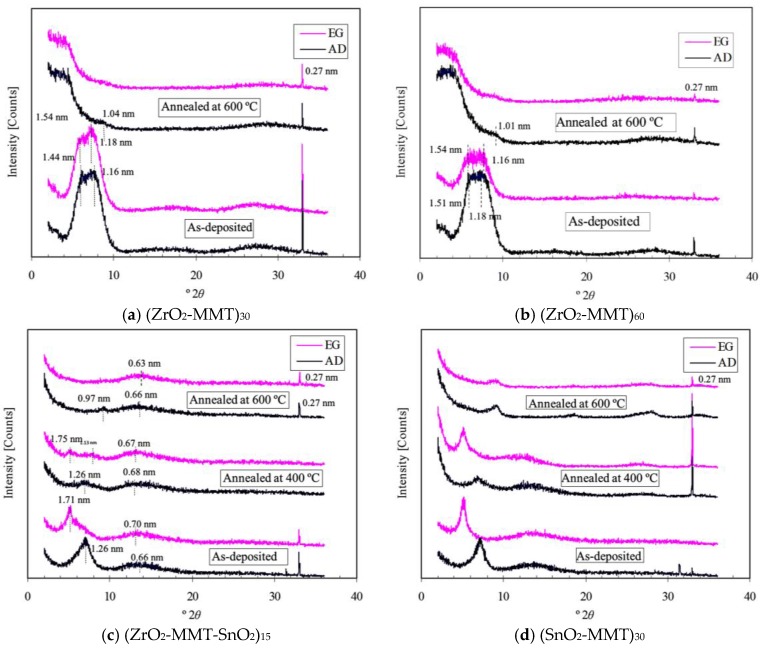
X-ray Diffraction (XRD) patterns of clay-oxide multilayers with clay concentration of 0.40 wt %. (**a**) (ZrO_2_-MMT)_30_ with Na-HMP (5 g/L) to exfoliate the clay particles; (**b**) (ZrO_2_-MMT)_60_ with Na-HMP (5 g/L) to exfoliate the clay particles; (**c**) (ZrO_2_-MMT-SnO_2_)_15_; and (**d**) (SnO_2_-MMT)_30_.

XRD patterns of as-deposited and annealed multilayers (ZrO_2_-MMT)_30_, (ZrO_2_-MMT)_60_, (ZrO_2_-MMT-SnO_2_)_15_, and (SnO_2_-MMT)_30_ under air-dried (AD) and ethylene glycol-solvated (EG) preparation were obtained to study their expendability versus forming conditions as shown in Figure 10a–d.

The similar XRD patterns of (ZrO_2_-MMT)_30_ and (ZrO_2_-MMT)_60_ under as-deposited and annealed at 600 °C conditions shown in Figure 10a,b indicates that preparation of multilayers with consistent structures is achievable. Both as-deposited films have a broad peak region with the basal spacing from ~1.16 nm to ~1.54 nm should result from the montmorillonite with hydrated interlayer cation Zr^4+^ at different degrees of hydration, which agrees well with the commonly recognized d(001) spacing of around 1.0–2.0 nm. The presence of montmorillonite in these multiple layers demonstrates that some clay particles were undesirably adsorbed during the process of dipping in the clay suspension. In particular, no visible peak related to the periodic thickness formed by each deposition cycle for multilayers is observed, however, which appeared in clay-zirconia multilayers prepared with clay concentration of 0.03 wt % by Chen et al. [15]. Since the clay concentration used to prepare the above samples is 0.4 wt %, it manifests that it is necessary to determine a proper clay concentration in order to fabricate a clay-based multilayer with consistent periodic layered structures. Highly amorphous structures were observed from the XRD patterns of both annealed samples, which is not as expected as that of the clay concentration of 0.03 wt % reported by Chen et al. [15]. This further proves the importance of clay concentration in preparation of clay-based nanostructured multilayers.

Figure 10c shows the XRD patterns of (ZrO_2_-MMT-SnO_2_)_15_ under as-deposited and annealed at 400/600 °C conditions. Air-dried and ethylene glycol-solvated as-deposited samples exhibit two relatively sharp peaks at 1.26 nm and 1.71 nm, respectively, verifying the existence of clay particles adsorbed in clay-based multilayers again. This finding can also be obtained from the XRD patterns of the annealed samples (at 400 °C), which have weak peaks, but with almost the same values as those of as-deposited samples at less than 10° (2θ). It is further inferred that the expandability of montmorillonite with hydrated interlayer cations Zr^4+^ and Sn^4+^ is reversible even after being annealed at 400 °C. However, the corresponding peaks disappear in the XRD patterns of the annealed (at 600 °C) samples, probably due to the growth of ZrO_2_ and SnO_2_ nanocrystals, which disordered the multilayer structures [15].

The XRD patterns of (SnO_2_-MMT)_30_ shown in Figure 10d have almost the same features as those of (ZrO_2_-MMT-SnO_2_)_15_ except that two nearly identical peaks at 0.96 nm (AD) and 0.98 nm (EG) are observed. They may result from the consistent periodic deposition layers rather than montmorillonite particles, because montmorillonite particles should be expanded under ethylene glycol-solvated preparation. If these peaks are assumed due to montmorillonite particles, the only interpretation is that SnO_2_ nanocrystals formed under annealing temperature 600 °C contribute to the non-expandability of the formed clay-oxide multilayers. Based on this explanation, the multilayers (SnO_2_-MMT)_30_ after being annealed at 600 °C, should have better periodic layers than (ZrO_2_-MMT)_30_. However, this problem should be investigated more clearly in further studies.

## 4. Discussion

### 4.1. Analytical Method

The multilayers formed by the LbL procedure have intrinsic anisotropic properties. Since there is no theory applicable to the determination of mechanical properties of such micro- to nano-meter thick films, presently, the analytical method used in this paper is just an approach to approximately estimate the true film hardness and modulus in the direction perpendicular to the indentation surface, in which the main purpose is to use these mechanical parameters as references to check multilayer design variables and compare different film-forming conditions. The hardness defined by the applied load divided by the corresponding projected area is the mean contact pressure and is found to be proportional to the film’s yield or flow stress in compression for isotropic materials [34]. The hardness of multilayers has a similar physical meaning if the same concept is assumed valid. The difficulty is the interpretation of the modulus of the multilayer determined by the nanoindentation test. This is mainly due to the complex stress and strain distributions generated beneath the indenter and the continuously changed boundary conditions as the indenter penetrates into the sample. However, the modulus determined by the above method could be a good comprehensive parameter to characterize the elastic properties, because it is obtained based on the elastic response of the film during unloading. The true modulus normal to the loading surface is believed to be related to the measured modulus with some extent of degree-dependence on the anisotropy of the film. This correlation is unclear and needs to be studied further.

Another concern are the errors due to pile-up mainly observed in soft films on hard substrates (e.g., as-deposited multilayers in this paper). The main reason is that the contact depth *h_c_* in Equation (2) is determined based on an elastic contact analysis without consideration of plastic deformation occurring in elastic-plastic materials. The ignorance of pile-up will result in an underestimation of the contact area and, subsequently, an overestimation of hardness and modulus. For example, the hardness and modulus obtained for as-deposited multilayers in this paper, which exhibit pile-up around the contact impressions, should be the upper bound value.

### 4.2. Multilayer Design

**Figure 11:**
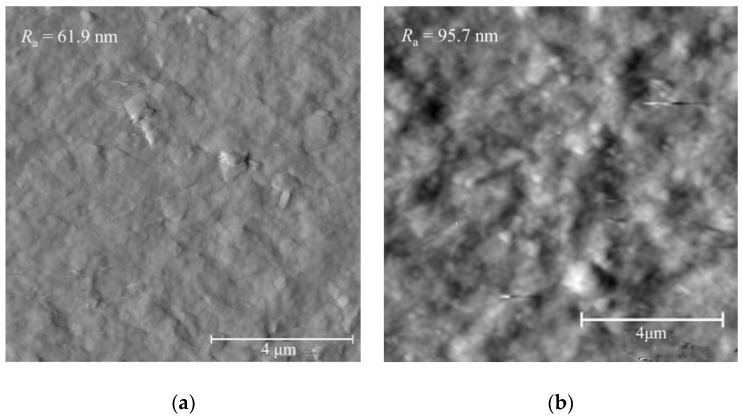
Surface roughness of (**a**) as-deposited and (**b**) annealed (at 600 °C) (ZrO_2_-MMT)_30_ with clay montmorillonite 0.4 wt %.

This paper focuses on the mechanical behavior of multilayers formed by the LbL deposition process under different conditions. The factors affecting on the mechanical properties mainly include cationic precursor, anionic precursor, dipping control parameters, and post-deposition treatment measures, which are the primary design parameters to achieve the expected requirements. Among these factors, the anionic precursor, exfoliated clay platelets (montmorillonite) in this paper, is the most difficult control parameter because of the inability to effectively characterize the exfoliation status. The unexfoliated clay aggregates could coexist with exfoliated clay platelets in the suspension, and the exfoliated clay platelets are also possible to reaggregate as the dipping deposition cycle proceeds. Therefore, it is difficult to ensure the identical clay layer for each deposition cycle. Even in the same deposition cycle, the in-plane distribution of clay platelets and particles is not uniform, which results in an uneven surface of the deposited clay layer. The annealing process can significantly reduce the porosity of the multilayers by the shrinkage normal to the deposition surface, but cannot reduce the sizes of clay aggregates. The growth of the oxide crystals during the annealing process could also deform the clay layers by displacement [15]. The surface roughness presents the degree of such accumulative evenness of a certain number of depositing cycles as shown in Figure 11. The surface roughness in both cases is not in the same order of the individual clay platelet thickness (~1 nm), implying that an effective dispersing and exfoliating method of clay particles is needed to prepare the clay suspension to generate an uniform clay layer in thickness and the in-plane distribution for each dipping cycle.

## 5. Conclusions

Nanoindentation tests were conducted under load control mode on 18 clay-based multilayer samples, which were prepared under as-deposited and annealing conditions at 400 °C/600 °C with different precursor cations and multilayer structure. The modulus and hardness of each sample are extracted from the measured composite response of the sample-substrate system. The load-displacement curves and residual AFM images show that all of the multilayers exhibit a superior tolerance to mechanical strain because of the high lateral bonding strength of clay platelets and strong bonding strength between interlayers. In addition, annealing significantly enhances the resistance of the multilayers to penetration or plastic deformation.

A common tendency was found that the modulus and hardness of all clay-based multilayers prepared with lower clay concentrations have much higher values than those with higher clay concentrations. This is probably due to the higher porosity of the films generated by higher clay concentrations. Consequently the higher periodicity of the multilayers also reduces the annealing effects for property enhancement. Analysis of the results with two annealing temperatures indicates that a relationship between the annealing temperature and a corresponding hardness and modulus for a specific type of clay-oxide multilayers exist, and the optimal annealing temperature can be determined case by case with respect to each type of the clay-oxide multilayers.

Clay-oxide multilayers prepared by the LbL method have promising applications with the improvement in accurate control of clay exfoliation, clay concentration, and optimization of annealing temperatures. The elastic modulus determined by nanoindentation is a suitable parameter for characterizing multilayers even though there still exist some unsolved theoretical issues with respect to nanoindentation testing on anisotropic layered materials.

## Figures and Tables

**Table 1:** Summary of prepared clay-based multilayers, treatment method and mechanical properties.

Film	Clay wt %	Formed Condition	Film Hardness *H*_f_ (GPa)	Film Modulus *E*_f_ (GPa)
(ZrO_2_-MMT)_30_	0.03	As-deposited	1.82	67.06
	0.03	Annealed at 600 °C	6.29	119.90
(ZrO_2_-MMT)_60_	0.03	As-deposited	1.67	63.78
	0.03	Annealed at 600 °C	2.89	120.10
(ZrO_2_-MMT)_60_ *	0.03	As-deposited	1.26	32.35
	0.03	Annealed at 600 °C	7.19	113.10
(ZrO_2_-MMT)_30_	0.40	As-deposited	0.33	9.94
	0.40	Annealed at 400 °C	0.49	7.74
	0.40	Annealed at 600 °C	1.68	43.75
(ZrO_2_-MMT)_60_	0.40	As-deposited	0.39	9.36
	0.40	Annealed at 600 °C	1.86	40.69
(SnO_2_-MMT)_30_	0.40	As-deposited	0.38	8.80
	0.40	Annealed at 400 °C	1.67	77.20
	0.40	Annealed at 600 °C	0.77	9.09
(ZrO_2_-MMT-SnO_2_-MMT)_15_	0.40	As-deposited	0.43	6.20
	0.40	Annealed at 400 °C	1.32	19.4
	0.40	Annealed at 600 °C	1.50	30.72
(ZrO_2_)_30_	0.03	Annealed at 600 °C	10.30	118.60

Note: MMT denotes montmorillonite; * denotes the multilayers prepared in clay suspension settled 24 h before being used.
